# Unidirectional motion of C_60_-based nanovehicles using hybrid substrates with temperature gradient

**DOI:** 10.1038/s41598-023-28245-4

**Published:** 2023-01-20

**Authors:** Mohammad Kianezhad, Mehrdad Youzi, Mehran Vaezi, Hossein Nejat Pishkenari

**Affiliations:** 1 Civil Engineering Department, Sharif University of Technology, Tehran, Iran; 2 Department of Civil and Environmental Engineering, University of California Irvine, Irvine, USA; 3 Institute for Nanoscience and Nanotechnology (INST), Sharif University of Technology, Tehran, Iran; 4 Nanorobotics Laboratory, Mechanical Engineering Department, Sharif University of Technology, Tehran, Iran

**Keywords:** Nanoscience and technology, Physics

## Abstract

With the synthesis of nanocar structures the idea of transporting energy and payloads on the surface became closer to reality. To eliminate the concern of diffusive surface motion of nanocars, in this study, we evaluate the motion of C_60_ and C_60_-based nanovehicles on graphene and hexagonal boron-nitride (BN) surfaces using molecular dynamics simulations and potential energy analysis. Utilizing the graphene-hBN hybrid substrate, it has been indicated that C_60_ is more stable on boron-nitride impurity regions in the hybrid substrate and an energy barrier restricts the motion to the boron-nitride impurity. Increasing the temperature causes the molecule to overcome the energy barrier frequently. A nanoroad of boron-nitride with graphene sideways is designed to confine the surface motion of C_60_ and nanovehicles at 300 K. As expected, the motion of all surface molecules is limited to the boron-nitride nanoroads. Although the motion is restricted to the boron-nitride nanoroad, the diffusive motion is still noticeable in lateral directions. To obtain the unidirectional motion for C_60_ and nanocars on the surface, a temperature gradient is applied to the surface. The unidirectional transport to the nanoroad regions with a lower temperature occurs in a short period of time due to the lower energies of molecules on the colder parts.

## Introduction

Understanding the motion of molecules on surfaces is of great importance in developing nano-transportation systems on the surface^1^. Using experimental and computational studies, many researchers have tried to understand the surface motion of various molecules in the past decade^2–9^. The experimental tools such as scanning tunneling microscopy (STM) require highly specialized equipment for investigating the surface phenomena at nanometer scale^10,11^. On the other hand, computational methods permit us to evaluate the effect of different parameters on the surface motion of nano-materials with lower costs.

In the primary studies, it was shown that the nanocars experience a diffusive motion on the substrate at different temperatures^12,13^. Recent computational attempts are mostly focused on proposing a controllable nanocar as a nanocarrier in the delivery of molecules^14,15^. Rectilinearity of motion has been achieved in some investigations using an external agent such as temperature gradient^16^ or electrical field^17,18^. In another group of studies, the directional surface motion of molecules has been obtained using surface modifications^19,20^. Using a hybrid substrate of gold and silver, Nemati et al.^21^ demonstrated that, the motion of molecules could be restricted to gold regions, bringing forth the idea of nanoroads. In other studies, it has been illustrated that C60 and fullerene-based nanocars experience directed movements using a thermal gradient on the substrate^22–24^. Kianezhad et al.^25^ have also studied the unidirectionality of the motion of carbon nanotubes on a golden substrate by evaluating the effects of chirality, diameter, temperature, and length.

The boron-nitride and graphene monolayers are capable of forming surface ripples (up to 1 nm out of plane, surface deformation in z-direction^26^). As a result, boron-nitride and graphene sheets demonstrate a wavy structure which can affect the surface motions^27,28^. Considering the structural and electrical characteristics^29,30^, graphene sheets can be considered as a potential candidate for nanoscale transportation applications. Mofidi et al.^26^ have studied the effect of thermal vibrations of graphene and surface ripples on the dynamical behavior of C60. They realized that surface ripples could result in decreasing desorption temperature due to the increase of the fluctuations of the molecule.

Similar to the atomic structure of graphene, a boron-nitride sheet is composed of a hexagonal structure of three boron and three nitrogen atoms. The boron-nitrogen bond length is 1.44 Å^31^, which is quite similar to the carbon–carbon bond length of 1.42 Å^32^ in graphene. Based on their structural similarity, graphene and boron-nitride sheets can be combined to form a hybrid substrate for transportation purposes at the nanoscale^33^. Vaezi et al.^34^ studied the mechanism of the motion of nanovehicles on hexagonal boron-nitride. They indicated that nanovehicles obtain higher mobility on the boron-nitride compared to the metallic surfaces. In another study, they investigated the motion of a C60 molecule on a hexagonal boron-nitride monolayer using molecular dynamics and potential energy approach to evaluate the effect of temperature, where they showed that C60 has lower potential energy on boron-nitride^33^.

Controlling the motion of molecules on the surface plays important role in the surface phenomena such as chemical reactions and catalysis^35,36^. The directional motions of nanomaterial are beneficial in the fabrication of nanostructures on the surface through the bottom-up approach^37^. On the other hand, the nanocars have been synthesis to carry payloads on the surface^38^. These nanomachines have shown the potential of transporting cargoes on the surface^39^. As a result, the directed movement of these nano-vehicles are also useful for designing nano-scale transportation and delivery systems on the surface.

In the present study, we evaluated the motion of C_60_ and C_60_-based nanovehicles on the graphene/hBN hybrid substrates to steer the surface motion, using the molecular dynamics method and the potential energy analysis. An initial assessment of the surface motion of molecules is presented based on the variation of the C60 potential energy on the BN and graphene surfaces. Using molecular dynamics simulations, the effect of impurities is investigated, suggesting C_60_ inclines to remain on the boron-nitride as it is more stable. Afterwards, using a nanoroad of boron-nitride inside a graphene substrate, we try to control the motion of objects on the surface. Finally, a combination of boron-nitride nanoroad and the temperature gradient is used to further steer the motion.

## Methods

**Figure 1:**
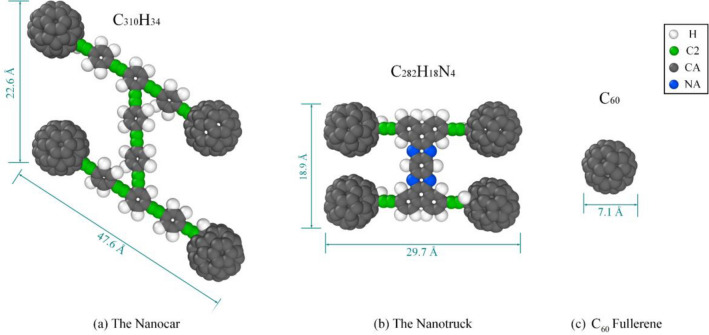
Schematic representation of (**a**) the nanocar, (**b**) the nanotruck, and (**c**) C_60_ fullerene. The molecular dimension is indicated for each molecule in the figure. A C_60_ has a molecular weight of $720.642 \frac{\mathrm{g}}{\mathrm{mol}}$, while the molecular weight of a C_60_-based nanocar and nanotruck is $3757.587 \frac{\mathrm{g}}{\mathrm{mol}}$ and $3602.202 \frac{\mathrm{g}}{\mathrm{mol}}$, respectively^22^.

In order to reach a comprehensive understanding of the dynamic behavior of nanomachines on the surface, we used both molecular dynamics simulations and potential energy analysis. In this study, the nanocar and the nanotruck results are compared with C_60_ since both are composed of four fullerene wheels. C_60_ is a rigid spherical molecule with I_h_ symmetry (Fig. 1c). It weighs $720.6$ g/mol, made of only carbon, and it is stable at high temperatures^40,41^. The primary difference between C_60_-based nanocar and nanotruck is the chassis^22^. The C_60_-based nanocar (Fig. 1a) is made of carbon and hydrogen, weighs $3757.587 \frac{\mathrm{g}}{\mathrm{mol}}$, and has C_2_ symmetry^42^. On the other hand, the chassis structure in a C_60_-based nanotruck (Fig. 1b) is different; besides carbon and hydrogen, there are four nitrogen atoms, making the chassis more rigid compared to the nanocar. The C_60_-based nanotruck weighs $3602.202 \frac{\mathrm{g}}{\mathrm{mol}}$ and it has a D_2_ symmetry which makes it more symmetrical than nanocar. Also, C_60_-based nanotruck is noticeably smaller than a nanocar^38^. Neither C_60_ nor nanocar and nanotruck has electric dipoles^21^. A schematic view of C_60_ and C_60_-based nanocar and nanotruck is indicated in Fig. 1, showing the atomic structure, molecular size, and chemical formulation of each molecule.

In this study, Tersoff potential^43^ has been employed to model the inner interactions of the graphene and boron-nitride monolayer. Also, substrate ripples are taken into account since the wavy surface can change the dynamical behavior of the nanocar on the graphene and boron-nitride monolayer due to the variation of contact level^29,34^. The internal interaction of fullerene is modeled using the Tersoff potential^43^ as well. Molecular mechanics (MM3) force field^44,45^ has been used to study the internal interactions of the C_60_-based nanocar and nanotruck. The bond and angle terms are considered by the harmonic style, while the dihedral style of OPLS force field was employed to calculate the torsional terms^46,47^. It is worth mentioning that improper terms are not included in the calculations. Two different types of carbon atoms are considered in the simulations as "$CA$" and "$C2$" based on their hybridization. "$CA$" atoms have sp^2^ or sp^3^ hybridization while "$C2$" carbon atoms have sp^34^. Different atom types are observable in Fig. 1. Supplementary material includes different parameters employed in the simulation of nanovehicles and fullerene.

Utilizing the Restricted Hartree–Fock (RHF) method, Vaezi et al.^34^ determined the partial atomic charge of each particle in the C_60_-based nanocar and nanotruck. Akimov et al.^17^ illustrated that with the absence of an external electric field, the charge transfer between non-bonded atoms is negligible. This conclusion has also been used in various studies^17,21–23,48,49^. As a result, we neglected the electrostatic forces to decrease the computational cost of the investigation since the charge transfer is not large enough to noticeably affect the motion. In order to account for the interactions between molecules (C_60_ and C_60_-based nanocar and nanotruck) with the graphene and boron-nitride monolayers, the 6–12 Lennard–Jones (LJ) potential with a cut-off radius of 13 Å is used. To be more specific, the LJ potential function employed to calculate the non-bonded interactions is as follow^50^:

1 $${E}_{LJ}=4\varepsilon \left({\left(\frac{\sigma }{r}\right)}^{12}-{\left(\frac{\sigma }{r}\right)}^{6}\right), r\le {r}_{cut}$$

where $\varepsilon$ and $\sigma$ are non-bonding parameters of LJ potential. Table S4 indicates the parameters used to model the non-bonding interactions. As shown in Table S4, identical LJ non-bonding parameters are used to model the interactions between the substrate and both carbon types in the nanovehicles^34,51–53^. Also, the non-bonding interactions between C_60_ and the substrate are considered the same as the “C2” (and “CA”) parameters, indicated in Table S4.

Noose–Hover (NVT ensemble) thermostat is employed to keep the desired temperature of the system constant. The temperature damping parameter of the thermostat (Tdamp), which determines the heat exchange rate between the thermostat and the system, is set to 100 fs. Then, before the primary simulation, the simulation system is allowed to relax freely at the desired temperatures for 1 ns to let the system reach an equilibration condition. The equations of motion were integrated using the velocity Verlet algorithm, and the time-step was set to 1 fs to achieve precise simulations. LAMMPS^54^ package was used for all the simulations.

The efficient application of nanomachines such as nanocars and nanotrucks requires a directional movement^20^. In this study, mean square displacement (MSD) is used to describe the diffusive motion of the surface-moving molecules. The diffusive coefficient is another parameter that can be derived from the MSD in order to characterize the diffusive motion. MSD is defined as^55^:

2 $$MSD\left(t\right)=<{|r\left(t\right)-r(0)|}^{2}>$$

where r is the center of the mass position vector. In statistical mechanics, <  > Expresses the ensemble averaging on the phase space, which is equal to the time integration of the system in ergodic terms^56,57^. In other words, <  > defines as averaging over all atoms. The slope of the MSD is defined as diffusion coefficient^55^.

3 $$D=\underset{t\to \infty }{\mathit{lim}}\frac{1}{bt}<{|r\left(t\right)-r(0)|}^{2}>$$

Since we are investigating the horizontal motion on the substrate in the XY plane, disregarding the vertical vibration, *b* is considered 4. However, for three-dimensional simulations, *b* will be 6.

For an accurate estimation of MSD, many simulations must be performed and then averaging should be done regarding the trajectory of all of them. Since this method is time-consuming, another way of calculating the MSD is to perform a relatively long simulation and then divide the simulation into smaller sub-trajectories. By assuming that each short-time trajectory is independent of others, averaging could be done over all of them^58^. In the present research, for MSD calculations, the data in each simulation is divided into 30 smaller simulations with 500 ps duration.

## Results and discussion

### The analysis of the potential energy

In this section, the potential energy between the molecules (i.e., nanocar, nanotruck, and C_60_) and the substrates have been evaluated. Considering the molecules and the substrate as rigid objects, the potential energy is calculated using the 6–12 Lennard–Jones potential. Additionally, because the results of this section are compared to those of molecular dynamics simulations, the initial configuration and the parameters of the Lennard–Jones potential are considered similar to those employed in the molecular dynamics analysis.

**Figure 2:**
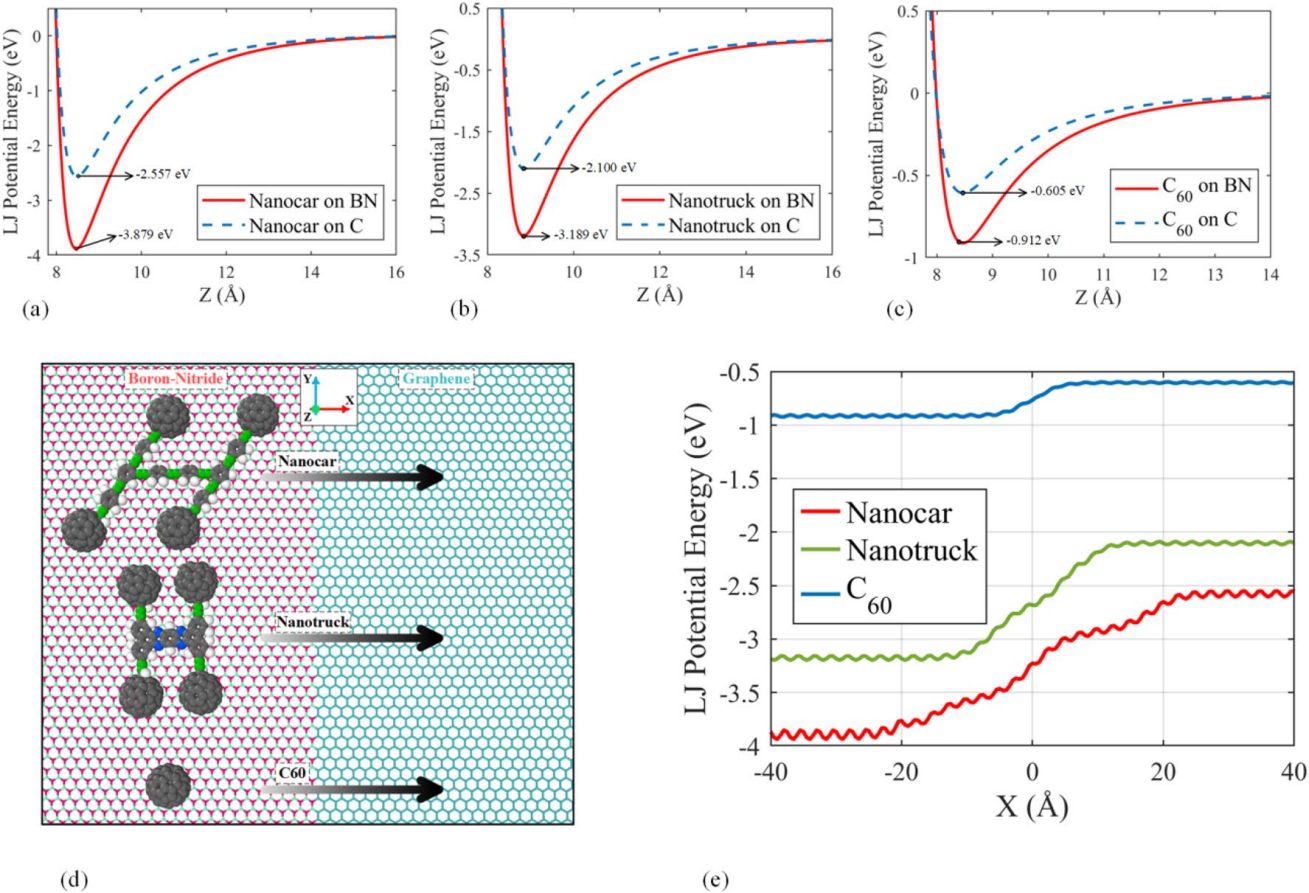
Potential energy as a function of the object's center of mass vertical distance from the substrate for (**a**) nanocar, (**b**) nanotruck, and (**c**) C_60_. The attractions between all objects and the pure boron-nitride substrate are stronger than those between objects and the pure graphene substrate. (**d**) Schematic representation of the setup considered for evaluation of potential energy when objects pass from pure boron-nitride substrate to pure graphene substrate. The coordinate system is illustrated in the figure. (**e**) The LJ potential energy captured when objects are passed from boron-nitride to graphene.

First, the intermolecular interactions between molecules and a pure boron-nitride and graphene substrate are measured separately, and their variation in Z-direction (Perpendicular to the substrate) is demonstrated in Fig. 2a–c. As depicted in Fig. 2a–c, the nanocars and fullerene experience more attraction on the pure boron-nitride substrate than on the pure graphene substrate. As a result, we can employ a substrate consisting of a mixture of both in some particular patterns to limit the molecular movement on the substrate. Moreover, the interaction between the nanocar and the substrate is stronger than that of the nanotruck and the substrate. The interaction between the nanotruck and substrate is stronger than the interaction between the C_60_ and the substrate, which arise from the fact that the nanocar is composed of more atoms than that of the nanotruck, and C_60_.

In the next step, to further survey the variation of potential energy, we investigated the potential energy in the case when objects are transferred from the pure boron-nitride substrate to the pure graphene substrate. The schematic representation of the setup considered for this evaluation is depicted in Fig. 2d. Each object is located on the boron-nitride side at first, and then by 0.5 Å steps, it shifts in the X-direction, and its minimum potential energy in Z-direction is computed. It is worth mentioning that, the vertical distance corresponding to the minimum potential energy is computed using the line search algorithm, and the minimum potential energy at the equilibrium point is recorded. The result of the preceding explained process for all objects is illustrated in Fig. 2e.

In Fig. 2e, the potential energy decay at the middle of the diagrams shows the transition of nanocars and C60 over the boundary, which depends on the structure of the molecules. In case of smallest molecule (i.e., C_60_), we observe smaller variation of potential energy. On the other hand, the nanocar has the largest descent of the potential energy, and since its wheels pass the border separately, the variation of potential energy does not have a constant slope. The difference between the potential energies of molecules, when they are placed on the boron-nitride and graphene substrate is the energy barrier against the transition. The highest and lowest energy barriers are corresponding to the transitions of nanocar and C_60_, respectively.

**Figure 3:**
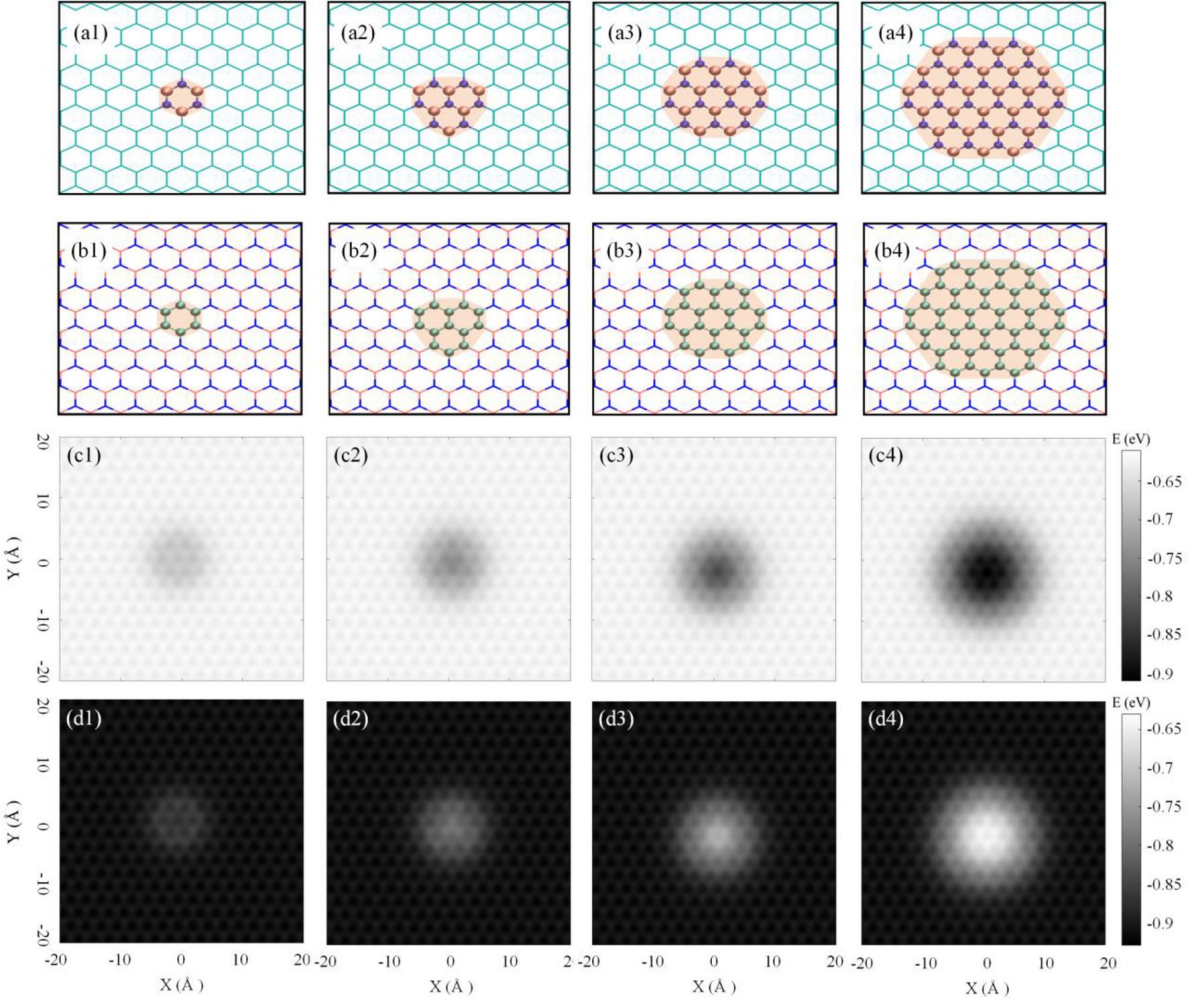
Impurities considered for analysis of potential energy. (**a**) A graphene substrate with 1, 3, 7, and 19 cells (hexagon) impurities of boron-nitride. (**b**) A boron-nitride substrate with 1, 3, 7, and 19 cells impurities of graphene. Each 1, 3, 7, and 19-cell impurity consists of 6, 13, 24, and 54 atoms, respectively. (**c**) The potential energy of the C_60_ during the horizontal displacement on the graphene substrate with boron-nitride impurities (a1, a2, a3, and a4 consist of 1, 3, 7, and 19 cells of boron-nitride impurities, respectively) and (**d**) boron-nitride substrate with graphene impurity (**b1**, **b2**, **b3**, and **b4** consist of 1, 3, 7, 19 cells of graphene impurities, respectively).

In the next step, eight types of local impurity are considered to evaluate the potential energy of C_60_ when it moves on these regions. Toward this aim, four circle-like impurities are considered in the middle of the graphene substrate, as shown in Fig. 3a. The impurity regions include 1, 3, 7, and 19 cells (hexagons) of boron-nitride, including 6, 13, 24, and 54 atoms of boron and nitrogen, respectively. Contrary to the former circumstance, the boron-nitride substrate with graphene impurities is also studied, whose schematic figures are depicted in Fig. 3b. The C_60_ was displaced horizontally in the X–Y plane with 0.1 Å steps, and the minimum potential energy in every point was calculated with respect to the vertical position of C_60_. It should be mentioned that, at each point, the hexagon face of the fullerene molecule is parallel to the surface. The final result is presented in Fig. 3c,d to clarify the effect of local impurity on the variation of potential energy.

The variation of the potential energy of C_60_ reveals that, increasing the size of boron-nitride impurity in the graphene substrate increases the depth of the potential energy well on the boron-nitride regions. As a consequence, the C_60_ requires higher thermal energy to overcome the energy barrier and move out of the impurity. On the other hand, as it can be seen in Fig. 3d, increasing the size of graphene impurity in the boron-nitride layer leads to a higher energy barrier in the graphene regions, which reduces the possibility of C_60_ entering this region. Also, it is presumable that C_60_ is unstable on the larger graphene impurity. In addition, the energy level in the center of 19 cell impurity is close to the state where the C_60_ is on the pure substrate. In other words, when C_60_ is located at the center of 19 cell impurity of both boron-nitride and graphene impurity, it almost does not interact with the atoms outside the impurity.

### Molecular dynamics of C_60_ on the local impurities

**Figure 4:**
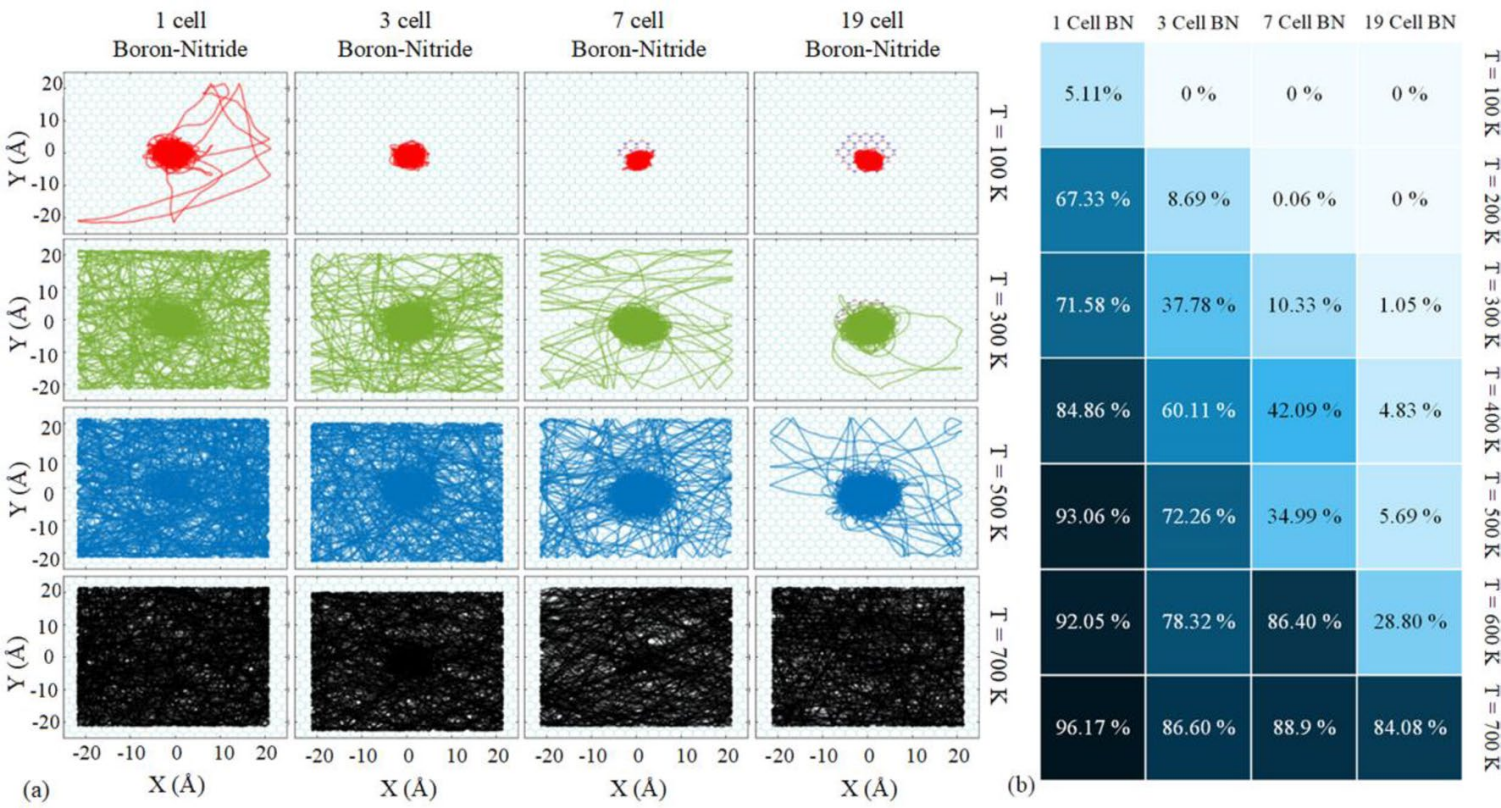
(**a**) Trajectory of C_60_ on the graphene substrate with a boron-nitride impurity at the center (to avoid the complexity of the figure, the trajectories at the temperatures of 100, 300, 500, and 700 K are only presented). (**b**) The portion of the whole simulation time in which C_60_ was located outside the impure region. Here, the graphene substrate has boron-nitride impurity in the center, as depicted in Fig. 3a.

Using molecular dynamics simulations, the trajectories of the motions of C_60_ on the substrates with the impurities are obtained (Fig. 3b). Each simulation is conducted in the temperature range of 100–700 K, with 100 K intervals. A 50 Å $\times$ 50 Å layer was considered as substrates, which includes the impurity at the center. After relaxation process, the atoms which are located at the edges of the substrate were considered fixed to prevent excessive distortion of the substrate. The trajectories of the motion of C_60_ on graphene substrate with 4 types of boron-nitride impurity are presented in Fig. 4, during the 15 ns simulations. Here, only the trajectories of the simulations at the temperatures of 100, 300, 500, and 700 K are plotted in Fig. 4a, while complete trajectories are available in the Supplementary material. It is noteworthy to mention that, the following trajectories are obtained from the position of the C_60_ center of mass (COM) relative to the substrate center of mass.

According to Fig. 4, C60 motion is completely restricted in all four impurities at 100 K, except for a short period of time in 1 cell impurity. At 300 K, the larger impurity limited the C_60_ motion on the substrate. In 500 K, the motion is not as limited as in lower temperatures except for 19 cells of boron-nitride impurity, where a noticeable limitation can be seen. However, at 700 K, the C_60_ receives sufficient thermal energy to overcome the energy barrier, and its trajectories do not show any restriction imposed by the impurity. The constraint movement of C_60_ induced by boron-nitride impurity is intensified by the size of the impurity and diminishes with the simulation temperature.

For a quantitative evaluation of the C_60_ motion on the graphene substrate, the simulation time corresponding to the motion of C_60_ on the graphene substrate was estimated, and by dividing it by the total simulation time, we obtained the portion of the simulation time in which the C_60_ was on the graphene substrate. Figure 4b approves that by increasing the temperature and decreasing the impurity size, the C_60_ is more likely to overcome the energy barrier of the impurity.

In the opposite state of the previous situation, Fig. S3 (see Supplementary material) exhibits trajectories of a C_60_ on the boron-nitride substrate with four types of graphene impurities at the temperature range of 100–700 K. As Fig. S3 illustrates, all four kinds of graphene impurity prevent the C_60_ to enter the impurity area, at the temperature of 100 K. At the temperature of 500 K, only large impurities noticeably prevent the C_60_ to enter the impurity regions (i.e., 7 and 19 cells). Eventually, the C_60_ can overcome the energy barrier and move on the graphene parts more frequently at the temperature of 700 K.

**Figure 5:**
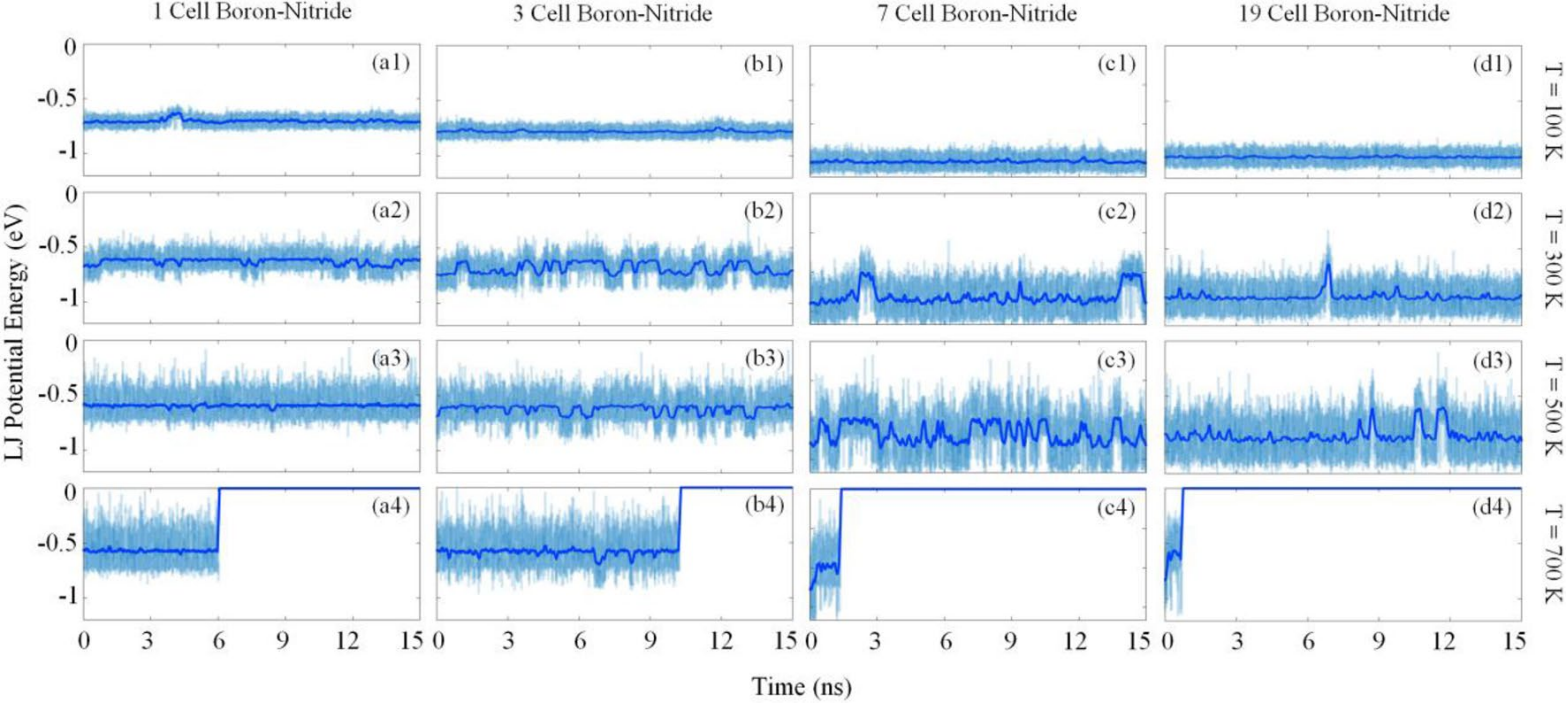
The Lennard–Jones interaction between C_60_ and graphene substrate with boron-nitride impurity. As presented, desorption has occurred in all impurity types at 700 K.

The intermolecular interactions between the C_60_ and substrate are studied during the molecular dynamics simulations. According to the potential energy between C60 and substrates (Fig. 5), the vdW interactions fluctuate around a constant level of energy when C_60_ remains on the boron-nitride impurity. In case of 1 cell impurity, the C_60_ is able to overcome the energy barrier for a short period of time, at 100 K. As a result, the potential energy level increases when the fullerene leave the boron-nitride impurity, and the intermolecular interactions decreased. It can be seen in Fig. 5 that, by enlarging the impurity at a constant temperature, the average potential energy of the C60 decreases, which shows the stronger attraction of C_60_ and the larger BN impurities. This observation was mentioned earlier in the potential energy analysis. By raising the temperature in a particular impurity size, the range of the energy fluctuation increases based on the higher thermal fluctuations of the fullerene at higher temperatures.

Another point that can be deduced from Fig. 5 is that, desorption occurs at the temperature of 700 K. In other words, the thermal fluctuations of C_60_ increase to 0 eV, which means that C_60_ has a long distance from the substrate at 700 K. Since the distance of fullerene to the substrate becomes more than the cut-off radius, the interaction between the C_60_ and the substrate becomes trivial. According to the result of the simulations at 700 K, it seems that the impurity size has no effect on the desorption of C_60_ on the impurity, which can explain the irregular change of percentages observed in the last row of Fig. 4b.

The desorption temperature of C_60_ on the graphene substrate with 1, 3, 7, and 19 cells of boron-nitride impurity is also studied. Based on Fig. 2c, when the distance of C_60_ COM from the substrate becomes more than 14 Å, the LJ potential energy becomes zero. Therefore, the 14 Å vertical distance can be considered as a threshold at which the fullerene and the substrate no longer interact with each other. Accordingly, desorption occurs when the C_60_ distance to the substrate reaches this threshold and never interacts again during the simulation. To report the desorption temperature in this investigation, we increased the temperature of the simulations by 25 K intervals to obtain the lowest temperature that the C_60_ desorbs. The results of the mentioned process for the C_60_ on the boron-nitride impurities are presented in Table 1. Although there is no regular relationship between the impurity size and the desorption temperature, the desorption temperature corresponding to the 19-cell impurity has a slightly higher value indicating that higher thermal energy is required for the desorption of fullerene due to the higher attraction of 19-cell impurity.

**Table 1:** The desorption temperatures of C_60_ calculated for different sizes of boron-nitride impurity in the middle of the graphene substrate.

1 cell BN impurity (K)	3 cells BN impurity (K)	7 cells BN impurity (K)	19 cells BN impurity (K)
$600\pm 25$	$625\pm 25$	$600\pm 25$	$650\pm 25$

### Steering the surface motion by designing a nanoroad

**Figure 6:**
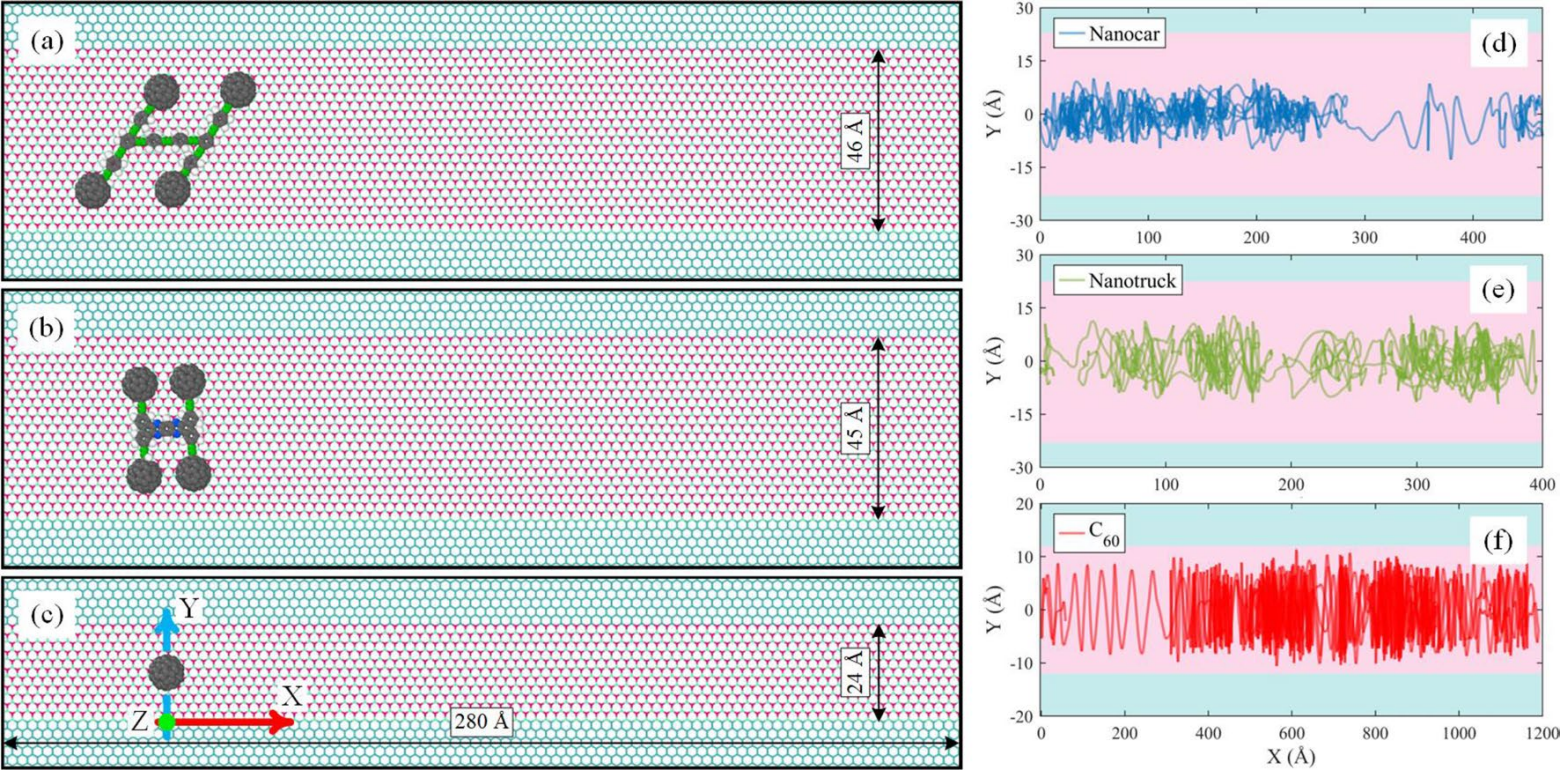
The initial condition of the simulation of a (**a**) nanocar, (**b**) nanotruck, and (**c**) C_60_ on the boron-nitride nanoroad. Different simulation systems were considered to study the unidirectionality of motion. Trajectories of (**d**) nanocar, (**e**) nanotruck, and (**f**) C_60_ on the nanoroad during 15 ns of simulation at 300 K. The blue and pink parts of each figure indicate the sections of graphene and boron-nitride substrate, respectively.

In this section, we aim to restrict the motion of nanocars and C60 in a unidirectional path by using the difference of the potential energy of the molecules on boron-nitride and graphene substrates. Therefore, both sides of boron-nitride substrate are bonded to the strips of graphene to limit the lateral movement of the molecules. The schematic representation of the initial conditions of the C_60_, nanocar and nanotruck on this particular type of substrate (i.e., nanoroads) are depicted in Fig. 6. The width of the boron-nitride part of the substrate is chosen proportional to the width of nanovehicles and fullerene. More precisely, the width of the boron-nitride part of the substrate is about 8.5 Å larger than the width of nanovehicles and C_60_.

As demonstrated in the simulation setup, the length of the nanoroad is 280 Å, and contrary to the Y-direction, the boundary condition of the nanoroad is periodic in the X-direction. The temperature of the simulations was adjusted to 300 K. The simulations were performed for 15 ns, and the nanovehicles and fullerene were allowed to move freely on the substrate. The trajectory of nanocar, nanotruck, and fullerene during 15 ns simulations are shown in Fig. 6d–f. The blue strips of the substrate in Fig. 6 indicate the graphene substrate, and the pink part in the middle indicates the boron-nitride nanoroads.

As expected, C60 and nanocars demonstrate limited motion in the Y-direction, and0 none of the molecules entered the graphene region. Although fullerene and nanovehicles oscillate in Y-direction, they experience directional movements in X-direction. As a result, using a nanoroad, a directional motion is available for molecules on the surface. Nonetheless, the molecules still have diffusive motion in the Y-direction, especially C_60_, which has less interaction with the substrate due to the fewer atoms of molecule.

### Unidirectional motion by applying temperature gradient on the nanoroads

In this section, to decrease the fluctuations of molecules and reach a more unidirectional motion, the effect of temperature gradient on the surface is surveyed. Since the free energy of the system decreases in the physical phenomena^59^, the nanocars and fullerene are expected to move from the beginning of the substrate with higher temperature to the colder end of the substrate.

**Figure 7:**
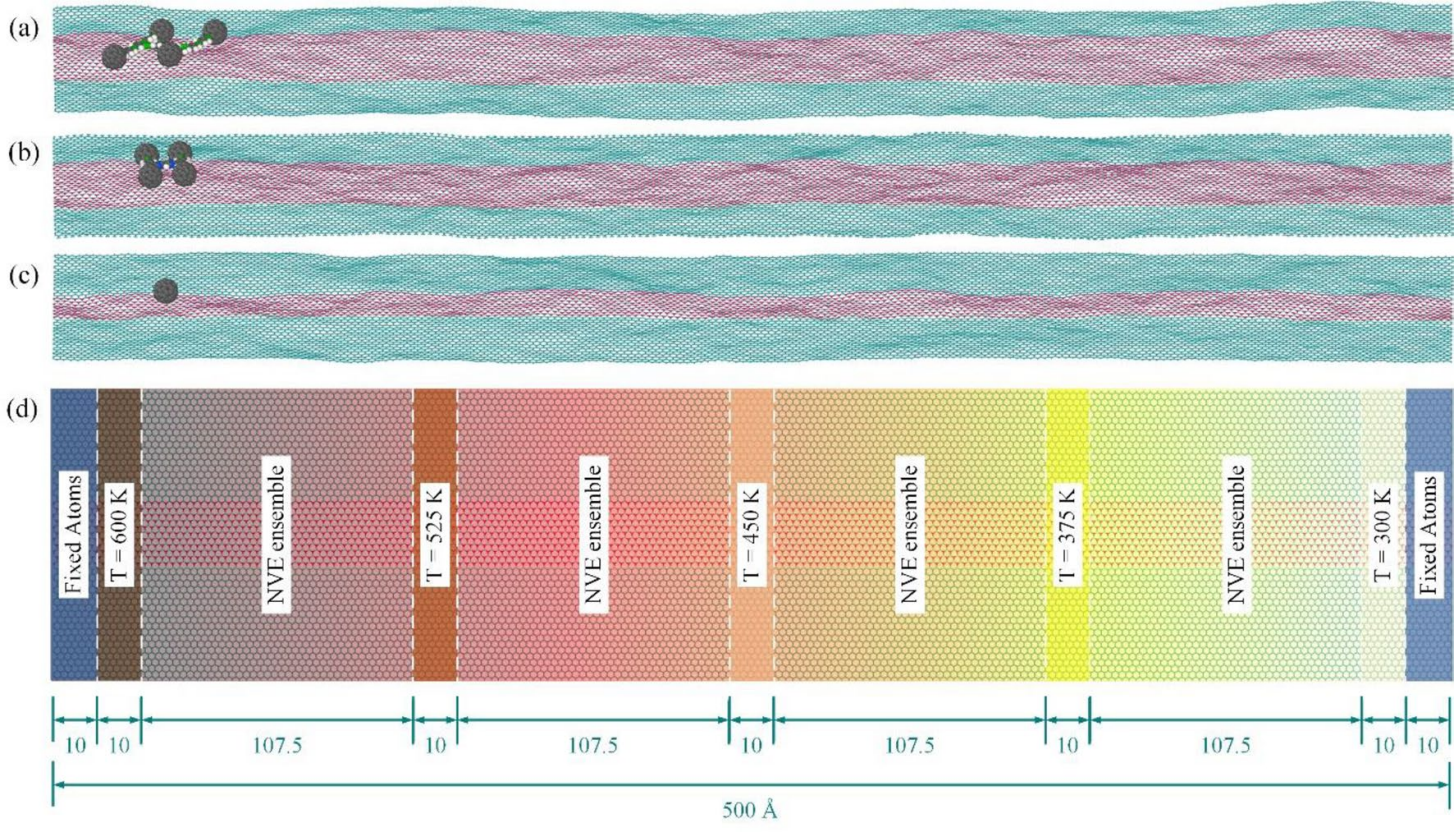
The simulation setup of (**a**) nanocar, (**b**) nanotruck, and (**c**) C_60_ on the nanoroad exposed to the thermal gradient. (**d**) Schematic view of the simulation setup, used to create a suitable thermal gradient along the substrate.

In order to create models with an appropriate uniform temperature gradient, the following setup is implemented. First of all, a similar substrate with a length of 500 Å is considered, as employed in the last section. The temperature of the left side and the right side of the substrate are considered 600 and 300 K, respectively. Then, the substrate is allowed to relax with an NVT ensemble at 450 K for 100 ps. Afterwards, as shown in Fig. 7, a 10 Å strips of the beginning and the end of the substrate is fixed to prevent large displacements of the substrate. Next, the NVT ensembles are employed to apply the temperature gradient, as demonstrated in Fig. 7. To obtain a regular thermal distribution, five NVT ensembles are applied to five groups of substrate atoms at equal distances. Also, the NVE ensembles are applied to other atoms between NVT groups. Finally, the system was relaxed for 1 ns to find the desired temperature gradient on the surface.

**Figure 8:**
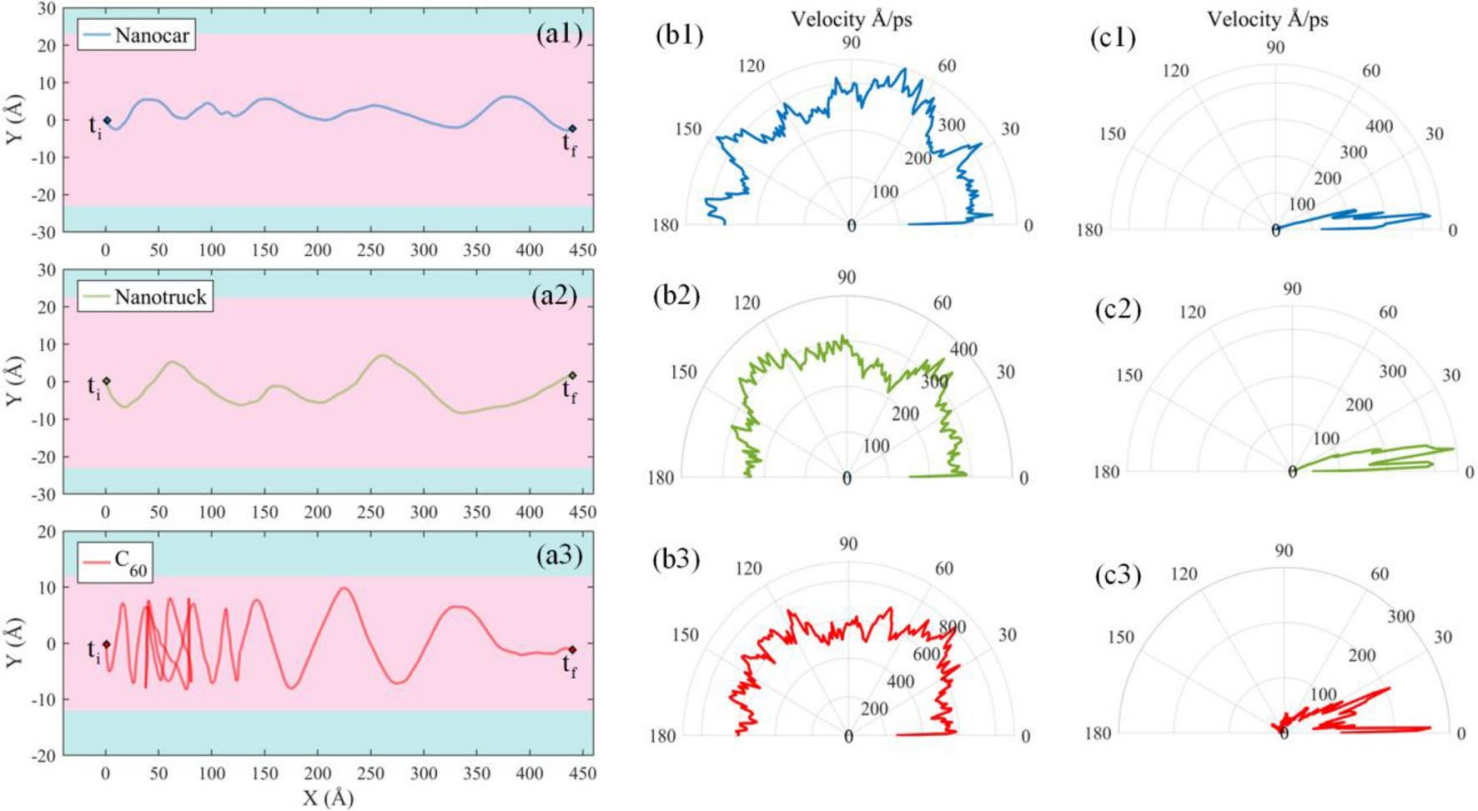
(**a**) Trajectories of nanocar, nanotruck, and C_60_ on the nanoroad exposed to the temperature gradient that changes from 600 K (left part of the substrate) to 300 K (right part of the substrate). The blue and pink parts of the figure indicate the graphene and boron-nitride sections of the substrate, respectively. The distribution of absolute values of velocity in different angles with respect to the X-direction for (**b1**) nanocar, (**b2**) nanotruck, (**b3**) C_60_ with the constant temperature, and for (**c1**) nanocar, (**c2**) nanotruck, and (**c3**) C_60_ with the temperature gradient. It is clear that the horizontal velocity of the molecule’s center of mass is more directional when the thermal gradient is applied to the substrate, and most of the velocity vectors are almost distributed around the positive direction of the X-axis.

The C_60_ and nanovehicles are placed at the left part of the substrate. The simulation continues until the COM of molecules reaches the right edge of the substrate with a temperature of 300 K. The trajectory of the motion of molecules is exhibited in Fig. 8 during the main simulations.

As we observe in Fig. 8, the fluctuation in the Y-direction is dramatically reduced for C_60_, nanocar and nanotruck compared to the simulations of previous section, in which the temperature was constant throughout the substrate. Because the nanocars and C60 tend to move toward the cold region on the right side of the substrate, most of their movements are in the X-direction.

In the following, a quantitative comparison is provided to better comprehend the influence of the temperature gradient on the surface motion of molecules. The average speed of the nanocar, nanotruck, and fullerene in the X-direction on the nanoroad with uniform temperature are 0.0314 Å/ps, 0.0263 Å/ps, and 0.0794 Å/ps, respectively. On the other hand, the average speed of these molecules in the X-direction on the nanoroad with the temperature gradient are 1.6734 Å/ps, 1.4982 Å/ps, and 0.8237 Å/ps, respectively. Accordingly, it can be seen that the average speed of the molecules in the X-direction has increased significantly in the presence of the temperature gradient compared to the motion at constant temperature. This is because the molecules spend less time oscillating in the Y-direction in the presence of temperature gradient. Correspondingly, nanocar, nanotruck and C_60_ tend to move in one direction, which increases the distance traveled in the same period of time (speed) in comparison with the motion of molecules at constant temperature. Although C_60_ has a higher average speed in the X-direction when subjected to a constant temperature compared to nanovehicles, its average speed in the X-direction is lower than the nanovehicles when it is subjected to the thermal gradient. This is due to the fluctuations of the C_60_ in the Y-direction; thus, the fullerene requires more time to pass the road.

To better investigate the directionality of the motion in both conditions of constant temperature and temperature gradient, the horizontal velocity of the molecules COM is calculated in each time step. The positive direction of the X-axis is considered as a reference, and the angle between the horizontal velocity vector and reference is obtained in each time step. For a particular angle of ${\theta }_{i}$, the magnitude of the velocity where the angle between the horizontal velocity vector and reference is equal to ${\theta }_{i}$ is accumulated. It should be noted that the angles between the velocity vector and the reference vector are between 0 and 180 degrees. Using the abovementioned process, the distribution of the horizontal velocities of molecules are obtained (Fig. 8b,c). According to Fig. 8b,c, unidirectional motion is not perceptible when the substrate is subjected to a constant temperature, and the velocity is distributed at different angles. Nevertheless, when a thermal gradient is applied to the substrate, the velocity vector is clearly directional, showing that the motion is toward the right side of the substrate where the temperature diminishes.

In the next step, we defined the unidirectional motion index (UMI) to check the unidirectionality of the motion quantitatively. UMI is defined as follows:

4 $$UMI= \frac{\mathit{max}\left({{\varvec{V}}}_{{\varvec{a}}{\varvec{c}}{\varvec{c}}}\right)-\mathit{min}({{\varvec{V}}}_{{\varvec{a}}{\varvec{c}}{\varvec{c}}})}{max({{\varvec{V}}}_{{\varvec{a}}{\varvec{c}}{\varvec{c}}})}$$

where ${V}_{acc}$ is the accumulated velocity vector at different angles. This parameter finds values between 0 and 1. When the horizontal velocity is uniformly distributed in different angles, the index is equal to 0, and when the velocity is limited to a smaller range of angles, the UMI index will be closer to 1. The obtained UMIs of C_60_ and nanovehicles are given in Table S5 of Supplementary material for both substrates with different thermal conditions. According to the UMI values provided in Table S5, the molecules have a higher UMI when the substrate is subjected to a temperature gradient, and their motions are more unidirectional compared to the motions on the nanoroads with a uniform temperature.

## Conclusion

To provide the controllable motion of molecules on the surface, we evaluated the motion of C_60_ and C_60_-based nanocar and nanotruck on graphene/boron-nitride hybrid surfaces, using MD simulations and the potential energy analysis. It has been shown that C_60_ and nanovehicles are more stable on the boron-nitride impurity in a graphene monolayer unless enough thermal energy is provided to prevail the energy barrier. On the other hand, the molecules are not stable on a graphene impurity in a boron-nitride substrate. Considering different sizes of impurity reveals that at a constant temperature, larger boron-nitride impurities are more capable of restricting the motion.

Considering the energy barrier between two monolayers, we have tried to restrict the motion using a nanoroad of boron-nitride with graphene sideways. As a result of the higher attraction of molecules with boron-nitride regions, the motion of C_60_ and nanovehicles was limited to the boron-nitride pathways; however, a diffusive motion was still dominant, especially in the lateral direction.

To further control the motion, a thermal gradient was applied to the nanoroads. The surface molecules showed unidirectional motion towards the lower temperature part of the substrate to reach lower free energy. The rectilinear motion of C60 and nanovehicles induced by hybrid surfaces exposed to the temperature gradient benefits targeted nano-transportation on the surface.

## Supplementary Information

Supplementary Information.

## Data Availability

The computational protocols used in this work, the input and output files, are available upon request from corresponding author.
