# Supplementary material for: Unidirectional motion of C60-based nanovehicles using hybrid substrates with temperature gradient
Source: Sci Rep. 2023 Jan 20;13:1100. doi: 10.1038/s41598-023-28245-4 (PMC9860030; doi:10.1038/s41598-023-28245-4)
Supplement: Supplementary file 1 — Supplementary Information. [file 41598_2023_28245_MOESM1_ESM.docx]

Unidirectional Motion of C_60_-based Nanovehicles Using Hybrid Substrates with Temperature Gradient

*Mohammad Kianezhad^1^, Mehrdad Youzi^2^, Mehran Vaezi^3^, Hossein Nejat Pishkenari^4*^*

1Civil Engineering Department, Sharif University of Technology, Tehran, Iran

2Department of Civil and Environmental Engineering, University of California Irvine, Irvine, United States

3Institute for Nanoscience and Nanotechnology (INST), Sharif University of Technology, Tehran, Iran

4Mechanical Engineering Department, Sharif University of Technology, Tehran, Iran

**Corresponding Author:** * Email: [nejat@sharif.edu](mailto:nejat@sharif.edu) Phone: +98 21 6616 5543

# **Supplementary material**

## **1. Parameters employed in the simulation of nanocars**

To model the interplay among atoms of the nanocar and the nanotruck, a Molecular Mechanics (MM) force field is employed. Bonds and angles terms are considered in the harmonic style as follows:

$E_{bond}=K_{b}{(r-r_{0})}^{2}$ (1)

$E_{angle}=K_{a}{(-{}_{0})}^{2}$ (2)

Where $K_{b}$, r, and $r_{0}$ in Eq. (1) represents the bond stiffness, the bond stiffness, and the equilibrium bond distance, respectively. Also, in Eq. (2), $K_{a}$, θ, and ${}_{0}$ represents the angle stiffness, angle, and equilibrium angle, respectively. In the continue, the dihedral term style is presented:

$E_{dihedral}=\frac{1}{2}K_{d1}{(1+cos)}+\frac{1}{2}K_{d2}\left( 1-cos2 \right)+\frac{1}{2}K_{d3}\left( 1+cos3 \right)+\frac{1}{2}K_{d4}(1-cos4)$ (3)

In Eq. (3) ϕ is the dihedral angle, and $K_{d1}$ to $K_{d4}$ are the torsion stiffness parameter. Noteworthy to mention that improper terms are neglected, and the parameters of this potential are calculated on the basis of an MM3 force field^1^, which are exhibited in Table S1 to Table S3.

Table S1: Bonds parameter used in the molecular mechanics force field

| Bonds parameters | | |
| --- | --- | --- |
| $\boldsymbol{K}_{\boldsymbol{b}}\boldsymbol{(}\boldsymbol{eV}/{\boldsymbol{Å}^{\boldsymbol{2}}}\boldsymbol{)}$ | $r_{0}(Å)$ | Description |
| 48.6652 | 1.212 | C2 C2 |
| 30.8837 | 1.313 | C2 CA |
| 25.1593 | 1.392 | CA CA |
| 14.35 | 1.101 | CA H |
| 34.596 | 1.260 | CA NA |

Table S2: Angles parameter used in the molecular mechanics force field

| Angles parameters | | |
| --- | --- | --- |
| $\boldsymbol{K}_{\boldsymbol{a}}\boldsymbol{(}\boldsymbol{eV}/{\boldsymbol{rad}^{\boldsymbol{2}}}\boldsymbol{)}$ | ${}_{0}$ | Description |
| 1.46619 | $\pi$ | C2 C2 CA |
| 1.34141 | ${2\pi}/3$ | C2 CA CA |
| 1.34141 | ${2\pi}/3$ | CA CA CA |
| 1.12304 | ${2\pi}/3$ | CA CA H |
| 1.34141 | ${2\pi}/3$ | CA CA NA |
| 1.34141 | $0.638 \pi$ | CA NA CA |

Table S3: Dihedral parameter used in the molecular mechanics force field

| Dihedral parameters | | | | |
| --- | --- | --- | --- | --- |
| $\boldsymbol{K}_{\boldsymbol{d}\boldsymbol{1}}\boldsymbol{(eV)}$ | $K_{d2}(eV)$ | $K_{d3}(eV)$ | $K_{d4}(eV)$ | Description |
| 0 | 4.34E-05 | 0 | 0 | CA C2 C2 CA |
| 0 | 4.34E-05 | 0 | 0 | C2 C2 CA CA |
| 0 | 0.650451 | 0 | 0 | C2 CA CA CA |
| 0 | 0.650451 | 0 | 0 | C2 CA CA H |
| -0.0403 | 0.208144 | 0 | 0 | CA CA CA CA |
| 0 | 0.234379 | 0.046 | 0 | CA CA CA H |
| 0.0433 | 0.650451 | 0 | 0 | CA CA CA NA |
| 0 | 0.390271 | 0 | 0 | H CA CA H |
| 0 | 0.650451 | 0 | 0 | H CA CA NA |
| 0 | 0.433634 | 0 | 0 | NA CA CA NA |
| 0 | 0.433634 | 0 | 0 | CA CA NA CA |

Table S4: LJ Non-bonding parameters employed to model the interactions between C_60_ and C_60_-based nanovehicles and substrate.

| Substrate | | Atom types of C_60_-based nanocar and nanotruck | | | | | | | |
| --- | --- | --- | --- | --- | --- | --- | --- | --- | --- |
|  |  | H | | C2 | | CA | | NA | |
|  |  | $\sigma(Å)$ | $\varepsilon(eV)$ | $\sigma(Å)$ | $\varepsilon(eV)$ | $\sigma(Å)$ | $\varepsilon(eV)$ | $\sigma(Å)$ | $\varepsilon(eV)$ |
| boron-nitride | B | 0.0017069 | 3.133 | 0.00329 | 3.411 | 0.00329 | 3.411 | 0.005085 | 3.409 |
|  | N | 0.0021086 | 3.089 | 0.00406 | 3.367 | 0.00406 | 3.367 | 0.006281 | 3.365 |
| graphene | C | 0.0013034 | 3.1065 | 0.0024 | 3.4 | 0.0024 | 3.4 | 0.0038825 | 3.3825 |

## **2. Supplementary results**

For a more comprehensive investigation, the motion of C_60_ will be studied through molecular dynamics method. First, the free motion of C_60_ on the pure boron-nitride and pure graphene substrate was simulated for 8 ns and in the temperature range of 100 to 500 K with 100 K intervals. Then, using the method mentioned in the methodology section, the diffusion coefficient for each simulation was calculated. The results of the motion C60 on pure graphene and BN surfaces are shown in Figure S1.

As shown in Figure S1, due to the fact that C_60_ has a more intensive attraction with the boron-nitride layer than the graphene layer, the diffusion coefficient of C_60_ on the boron-nitride substrate is less than the C_60_ on the graphene substrate in all temperatures. This implies that C_60_'s motion on the boron-nitride substrate is slower due to the stronger attraction between the fullerene and the boron-nitride monolayer. This result is appropriately in accordance with the results obtained in the potential approach.


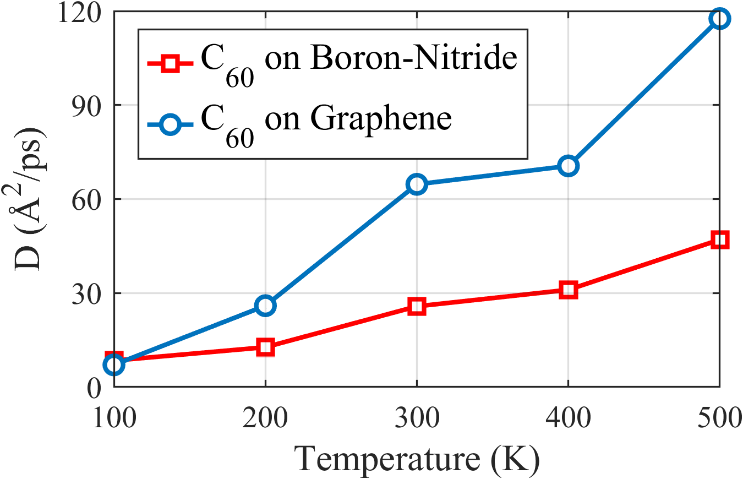


Figure S1: Diffusion coefficient of C_60_ motion on the pure boron-nitride and pure graphene substrate in 100 K to 500 K with a 100 K interval. At each temperature, the diffusion coefficient concerning C_60_ motion on the pure boron-nitride substrate is less than the C_60_ motion on the pure graphene substrate.


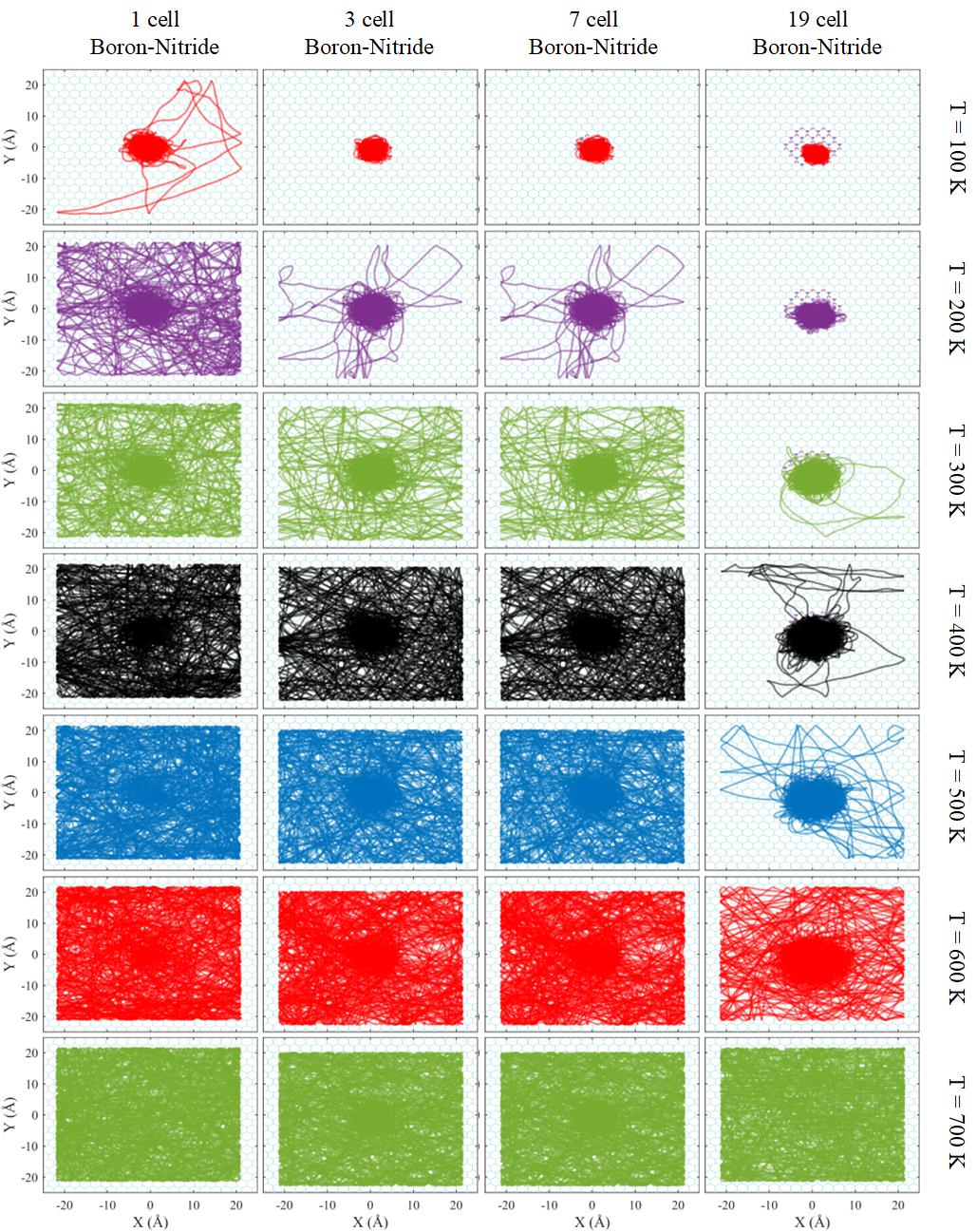


Figure S2: Trajectory of C_60_ center of mass relative to the substrate center of mass on the graphene substrate with a boron-nitride impurity at the center. Accordingly, the constraint of C_60_ movement enforced by boron-nitride impurity has a clear relationship with the size of the impurity and an inverse relationship with the simulation temperature.


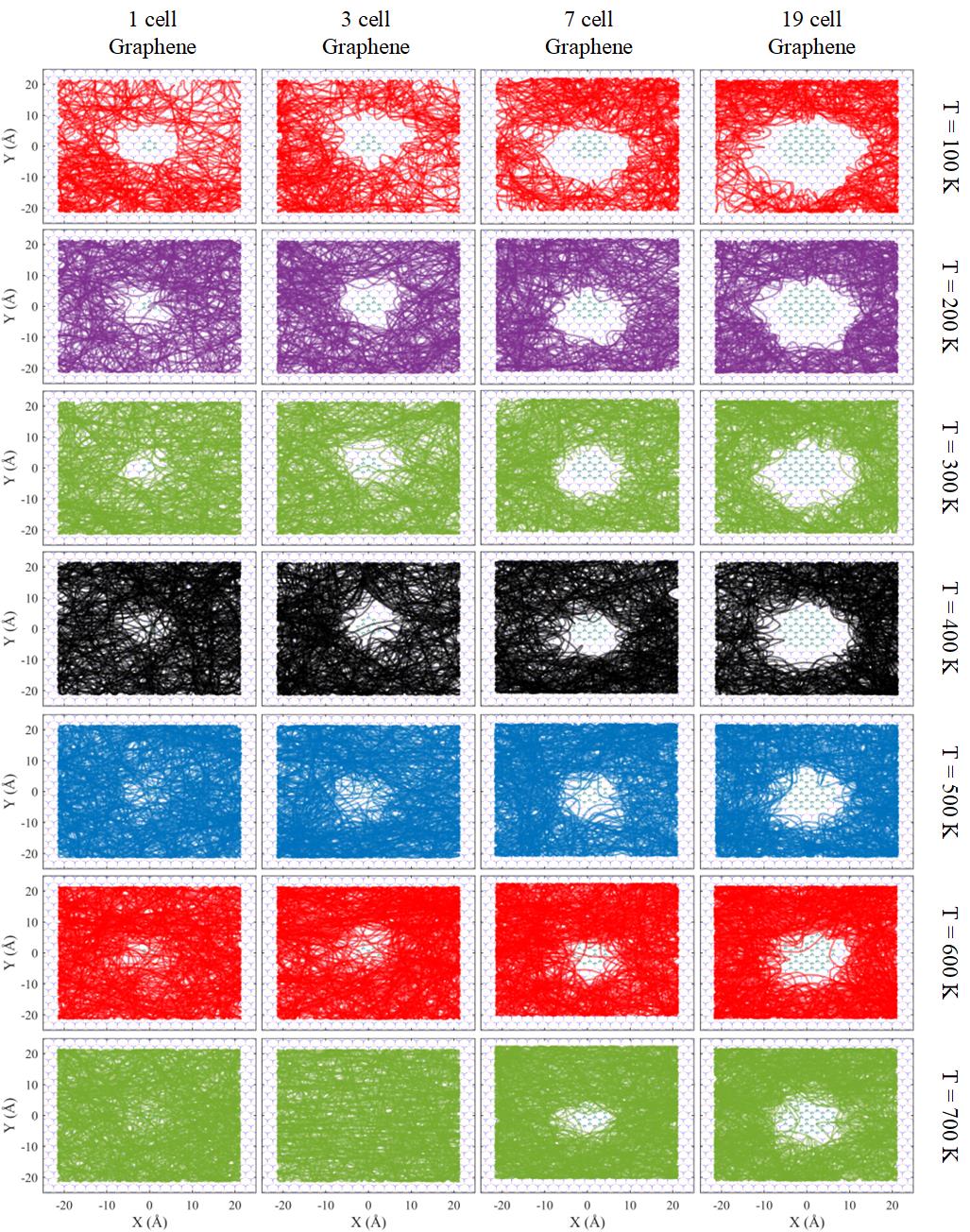


Figure S3: Trajectory of C_60_ center of mass relative to the substrate center of mass on the Boron-Nitride substrate with a graphene impurity at the center. Accordingly, the constraint of C_60_ movement enforced by graphene impurity has a clear relationship with the size of the impurity and an inverse relationship with the simulation temperature.

The MSD parameters of the nanocar, nanotruck, and C_60_ during the simulation were calculated and presented in Figure S4. As the C_60_ trajectory in Figure 6d-f implies, C_60_'s MSD is remarkably higher than the nanocar and nanotruck because of smaller intermolecular interaction. Also, due to the higher slope of the MSD curve of C_60_, we can conclude that the diffusion coefficient of C_60_ is higher than the other two nanovehicles. Therefore, as mentioned before, due to the high fluctuations of C_60_ in the Y-direction, it experiences more diffusive motion than nanocar and nanotruck, while the MSD and diffusion coefficient of nanocar and nanotruck are almost similar.


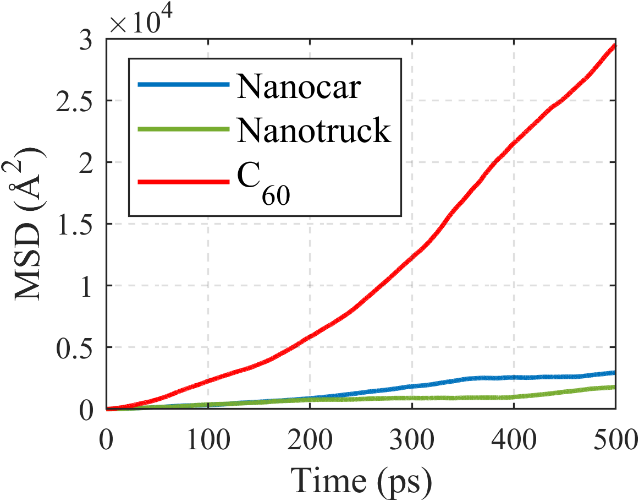


Figure S4: Mean squared displacement (MSD) of nanocar, nanotruck, and C_60_ during the simulations corresponding to the trajectories in Figure 6d-f.

In Figure S5, several snapshots of the motion of nanocar, nanotruck and fullerene on the nanoroad with temperature gradient are depicted. A closer look at Figure S5 indicates that, C_60_ requires more time to travel the road and reach the right end. This is due to its lower intermolecular interactions with the substrate, which cause the fullerene to fluctuate more frequently than the nanovehicles in the Y-direction.


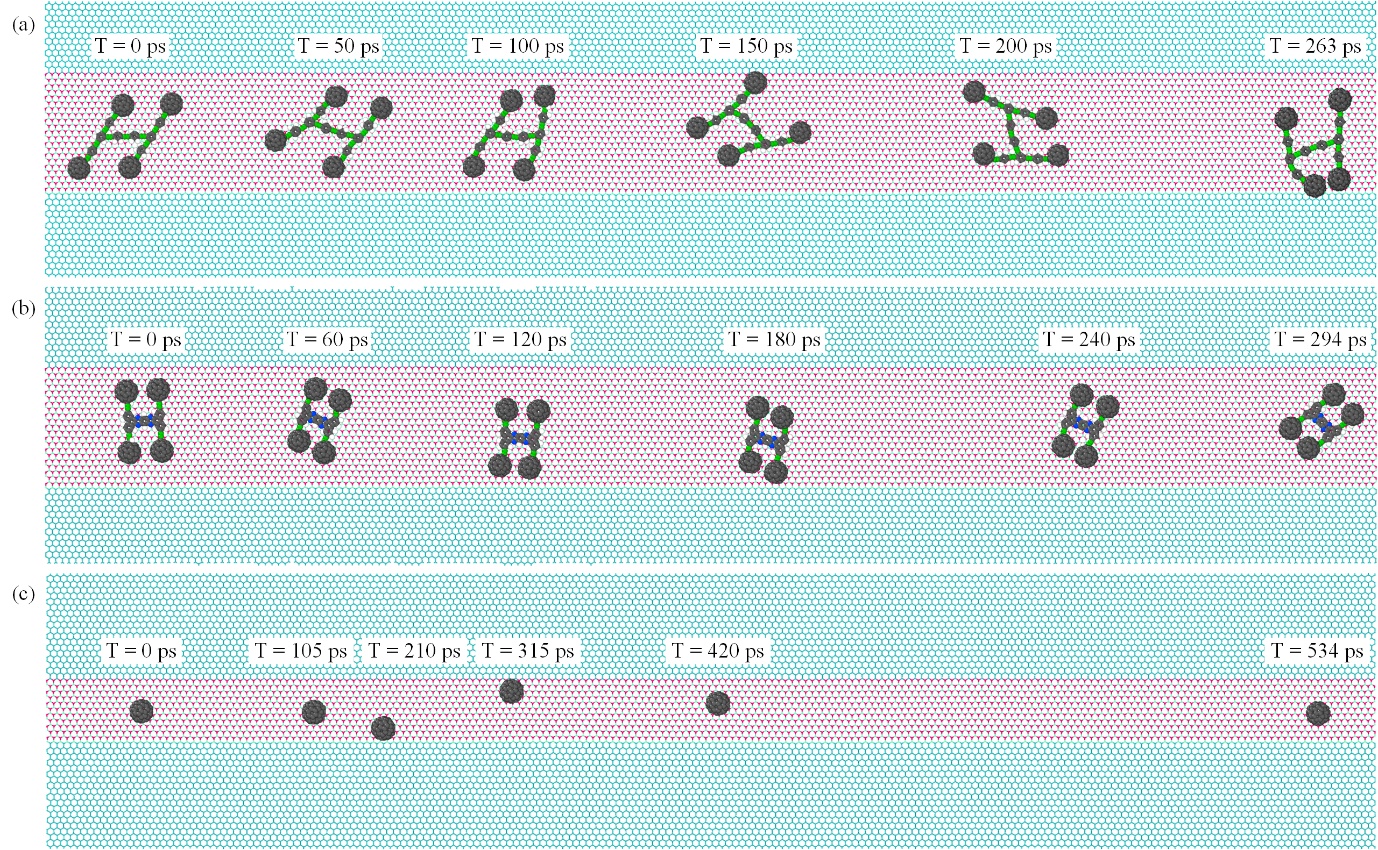


Figure S5: Snapshots of the motion of (a) nanocar, (b) nanotruck, and (c) C_60_ on the hybrid substrate with a temperature gradient that changes from 600 K to 300 K.

Table S5: The unidirectional motion index for nanocar, nanotruck, and C_60_ on the substrate with uniform temperature and with temperature gradient. The molecules on the substrate subjected to the temperature gradient have a more unidirectional movement.

|  | nanocar | nanotruck | C_60_ |
| --- | --- | --- | --- |
| With uniform temperature | 0.6578 | 0.5709 | 0.6782 |
| With temperature gradient | 1 | 1 | 0.9992 |

# **References**

1. Nemati, A., Meghdari, A., Nejat Pishkenari, H. & Sohrabpour, S. Investigation into thermally activated migration of fullerene-based nanocars. *Sci. Iran.* **25**, 1835–1848 (2018).
